# Lymphatic filariasis transmission 10 years after stopping mass drug administration in the Gomoa west district of Ghana

**DOI:** 10.1016/j.ijid.2025.107790

**Published:** 2025-03

**Authors:** Christian Akuamoah Boateng, Millicent Selassie Afatodzie, Angus McLure, Bethel Kwansa-Bentum, Dziedzom K. de Souza

**Affiliations:** 1 Department of Parasitology, Noguchi Memorial Institute for Medical Research, College of Health Sciences, University of Ghana, Accra, Ghana; 2 Department of Animal Biology and Conservation Science, School of Biological Sciences, College of Basic and Applied Sciences, University of Ghana, Accra, Ghana; 3 National Centre for Epidemiology and Population Health, The Australian National University, Canberra, Australia

**Keywords:** Lymphatic filariasis, Molecular xenomonitoring, Post-MDA surveillance, Ghana

## Abstract

**Objectives:**

A survey was conducted 10 years after stopping MDA in the Gomoa West District of Ghana to assess the *Wuchereria bancrofti* prevalence in both human and mosquito populations.

**Methods:**

In seven communities, infection in humans was assessed using the filariasis test strip (FTS). Mosquitoes were collected once a month over six months using pyrethrum spray catches (PSC). The mosquitoes were analyzed for *W. bancrofti* infections, using dissection followed by poolscreening PCR.

**Results:**

FTS results showed that 2/524 (0.38%; 95% CI, 0.0%-0.9%) individuals tested positive for antigen. Dissections revealed *W. bancrofti* infections in 5/107 *Anopheles gambiae* (4.7%: 95% CI, 2.2-8.5) from one community, with three mosquitoes harboring L3 larvae (2.8%: 95% CI, 0.9-7.5). PCR analysis of 683 mosquitoes in 57 pools revealed seven positive pools from two communities. The prevalence of infected mosquitoes by PCR for the district was 3.1% (95% CI, 0.5-24.0) for *An. gambiae* and 2.5% (95% CI, 0.4-23.5) for all *Anopheles* spp.

**Conclusions:**

The infection rate in the *Anopheles* spp. exceeds the provisional 1% threshold suggested by WHO, indicating ongoing transmission risk ten years after stopping MDA. Further district-wide assessments are recommended to inform the scope of any interventions required in the Gomoa West district.

## Highlights

• The Gomoa West District of Ghana stopped LF MDA in 2014.
• Antigen prevalence in the humans was very low, 10 years after stopping MDA.
• Pool screening infection in the vectors was higher than the WHO threshold of 1%.
• Molecular xenomonitoring is essential for post-validation surveillance efforts.

## Introduction

Having targeted the elimination of lymphatic filariasis (LF) as a disease of public health problem, many disease-endemic countries have been implementing mass drug administration (MDA) to at-risk populations to bring infection thresholds below levels where transmission cannot be sustained. Thus, since the launch of the Global Programme to Eliminate Lymphatic Filariasis (GPELF) [1] in 2000, 19 countries (including Togo and Malawi in Africa) were successfully validated for elimination [2]. 14 countries have stopped MDA in all endemic districts and are under post-MDA surveillance, while 33 countries have scaled MDA to all endemic districts [2]. There remains one country (Gabon) where MDA is yet to start, while in five others MDA is yet to be fully scaled to all endemic districts. In Ghana, MDA has been scaled to all endemic districts and stopped in 110 of the 118 districts as of 2022 (ESPEN database) [3]. However, an important aspect in ensuring successful elimination and maintaining the gains achieved is the need for effective surveillance systems to detect recrudescence or resurgence of infections and institute measures to address any challenges effectively [4]. Surveillance in both the human and vector populations is recommended.

The resurgence of LF following the cessation of MDA in some previously endemic communities is not new [5,6]. In Ghana, numerous studies have examined the impact of MDA during active intervention periods [7,8], but there is a relative paucity of data on transmission following the cessation of MDA. WHO recommends ongoing surveillance in previously endemic areas to monitor for signs of recrudescence and guide necessary interventions [4]. This study was therefore conducted in the Gomoa West District, where MDA has been stopped since 2014 following successful transmission assessment surveys, to assess the prevalence of infection in the human and mosquito populations.

## Methods

### Description of study sites

**Figure 1:**
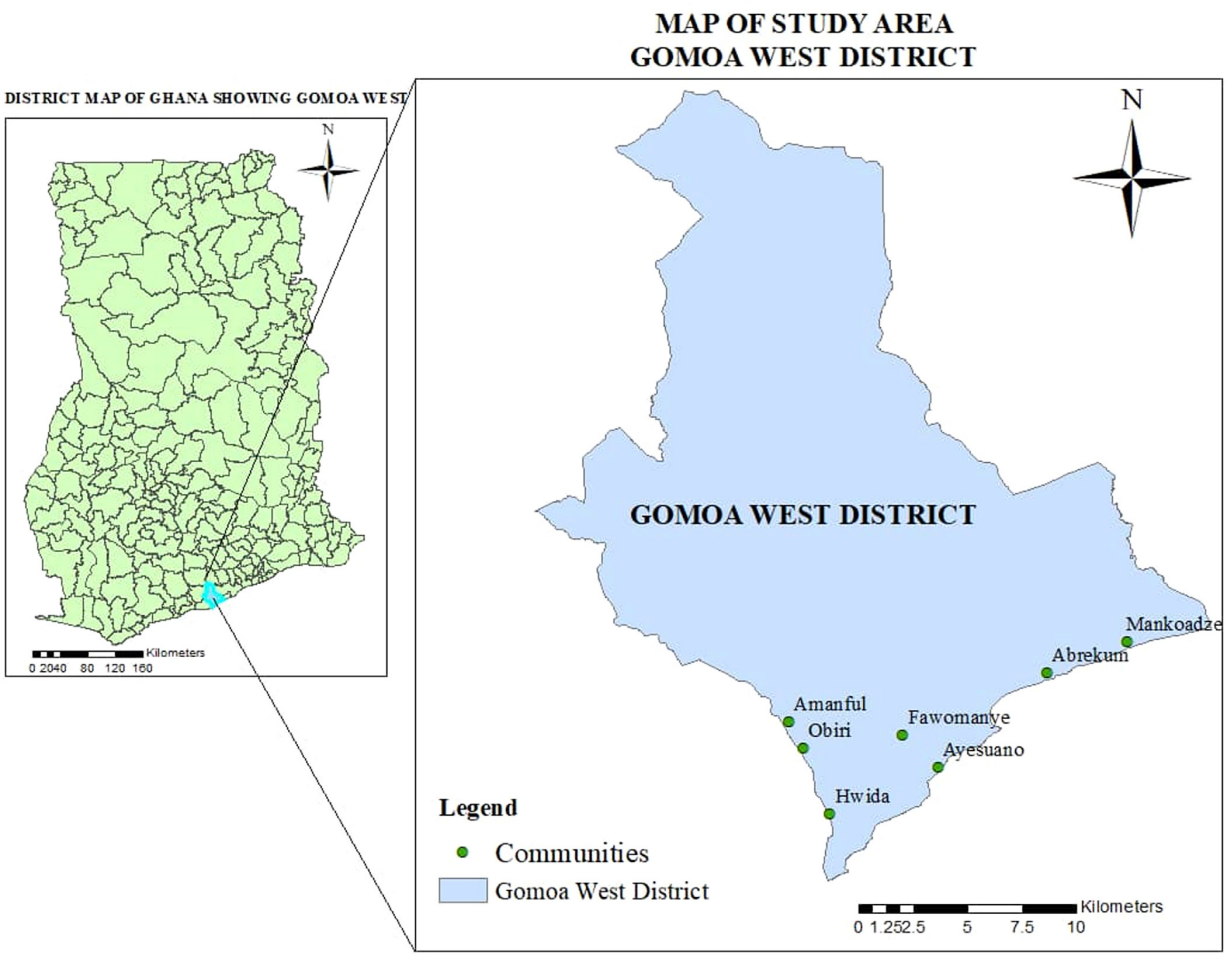
Map of Gomoa west district showing the location of study communities.

The study was conducted in the Gomoa West District in the Central Region of Ghana, located along the coastal savannah area, 50 km west of Accra. Seven communities were selected for the study (Figure 1). All the communities were previously located in the Gomoa district. However, following redistricting in 2008, the Gomoa district was divided into two districts including the Gomoa West district where the current study was conducted. Other than Abrekum, all the study communities were selected for monitoring activities based on previously high endemicity and availability of baseline data in these communities. As such the selection of the communities was purposeful to enable a comparison of the pre-MDA data and the findings from this current study. Before the implementation of MDA in the Gomoa district, the prevalence of microfilariae in the population was 4.6% (43/941) while CFA prevalence was 8.7% (75/861) [9]. The last MDA was conducted in 2014 in the Gomoa West and other adjoining districts. TAS3 results in the Gomoa West district in 2019 indicated one positive out of 1558 children tested (ESPEN database) [3]. The adjoining districts have also stopped MDA.

### Study design

This study employed cross-sectional screening of residents as well as entomological surveys conducted over six months in the seven study communities in the Gomoa West district.

### Community screening for *Wuchereria bancrofti* antigenemia and microfilariae

At least 50 participants were targeted from each community as per the WHO LF mapping protocol [10]. to account for refusals a 10% nonresponse rate was added to the sample size. Thus, a minimum of 55 participants per community (385 for the entire study) were targeted. The communities were notified a day in advance through local information centers and community leaders to gather at a designated location (usually the community center). Individuals, 18 years and above, were targeted for the study, informed, and if consented, taken through the study procedures. Demographic information was gathered from participants including age, gender, and occupation. Data was also gathered on previous MDA participation. The Filariasis Test Strip (FTS) was used for the qualitative detection of *W. bancrofti* antigenemia of participants during the day [11]. Briefly, a 75-microliter blood sample was collected from each participant via finger prick into a micropipette. The blood was transferred onto the FTS and the result read after ten minutes following the manufacturer's guidelines.

In antigen-positive individuals, a trained phlebotomist collected venous blood into EDTA tubes between 9 pm and 11 pm for microfilaria (mf) quantification. 100 µl of the blood sample was added to 9ml 3% acetic acid in an Eppendorf tube. The content was thoroughly mixed and then poured into a Sedgewick counting chamber. Counting of mf was then carried out under X10 magnification of a light microscope [12].

### Mosquito collection and processing

Mosquitoes were collected over 6 months from August 2023 to January 2024 as part of monthly entomological surveys that were carried out in each of the seven study communities. Each community was divided into quadrants and households were randomly selected from each quadrant for mosquito collection. In each community, indoor resting mosquitoes were sampled one day in a month between 5 am and 8 am, from 25 households in one quadrant, using the Pyrethrum Spray Catch (PSC) method. A total of 150 households were sampled per community. In each household, three rooms were randomly selected and sprayed. The dead and immobilized mosquitoes were collected and placed on wet filter paper lining labeled Petri dishes. The number of people sleeping in each room was recorded as a proxy to estimate biting rates and other entomological indices. The collected samples were transported to NMIMR Parasitology Laboratory for identification and processing.

The collected mosquitoes were identified to the species level using the morphological keys of Gillies and De Meillon [13]. The female mosquitoes were dissected into three parts (head, thorax and abdomen). A drop of 1% saline solution was added to each part and examined for L1, L2 and L3 stages of *W. bancrofti* under a dissecting microscope.

### DNA extraction and PCR for *W. bancrofti* identification

The dried carcass of dissected mosquitoes together with any *W. bancrofti* larvae found on each slide were scraped off into a 1.5 ml Eppendorf tube. Dissected mosquitoes in which any of the *W. bancrofti* larval stages were detected were processed singly to confirm the infection. All other mosquitoes were processed in pools of up to 20 mosquitoes. The Quick-DNA/RNA Pathogen kit (Zymo) was used in the extraction of pooled mosquitoes for parasite detection following the manufacturer's recommended protocol. The eluted DNA was frozen at -20°C until used for PCR. The PCR for the identification of *W. bancrofti* followed the conventional PCR method by Ramzy et al. [14].

### Genomic DNA extraction and molecular identification of *An. gambiae* s.l. mosquitoes

Genomic DNA was extracted from the legs of selected *Anopheles gambiae* s.l. morphologically identified, using the Quick-DNA/RNA kit (Zymo) following the manufacturer's protocol. The extracted DNA was used for PCR following the methods of Fanello et al. [15]

### Data analysis

The data was analyzed to determine the prevalence of antigenemia, and the parasitemia (mf) of participants, as well as the infection and infectivity of the mosquito species collected. Microsoft Office Excel spreadsheet was used to enter the questionnaire survey data, which was then imported into STATA version 17 (STATA Corporation, Texas, USA) for analysis. The PooledInfRate [16,17] package in R software version 4.3.3 [18] was used to estimate the prevalence of PCR-positive mosquitoes for each species and community. Point estimates included Firth's bias correction while 95% confidence intervals were computed using the score method, with adjustment for skewness where one or more pools were positive. For estimating prevalence at the district level, the HierPoolPrev function in PoolTestR [19] was used to account for clustering within communities, using default uninformative priors and the posterior median as the point estimate.

Entomological parameters were calculated as follows:

■ Infection rate: [Number of mosquitoes carrying *W. bancrofti* larvae of any stage (L1–L3)] **/** [Total number of mosquitoes dissected] x 100%.
■ Infectivity rate: [Number of mosquitoes carrying the infective L3 stage of *W. bancrofti*] **/** [Total number of mosquitoes dissected] x 100%.
■ Monthly biting rate (MBR): [Number of blood-fed mosquitoes captured] **/** [Number of sleepers] x 30.5.
■ Annual biting rate (ABR): [Number of blood-fed mosquitoes captured] **/** [Number of sleepers] x 365.
■ Annual infective biting rate (AIBR): ABR x Infectivity rate.
■ Worm load: [Total number of L3 **/** Total number of mosquitoes carrying L3].
■ Annual transmission potential (ATP): AIBR x Worm load.

## Results

### Epidemiological surveys

This study recruited a total of 524 participants across the seven communities. Except in Mankoadze and Ayesuano where 44 and 51 participants were recruited respectively, the sample size was met in the other communities due to the demand and the number of people who turned up (Table S1). 42.0% (220/524) of participants were males and 58.0% (304/524) were females. Overall, most participants (21.0%) were from the age group of 20-29 years with the smallest group (12.0%) in the 50-59-year group. Testing with the FTS showed that 2/524 (0.38%; 95% CI, 0.05%-1.37%) individuals were positive for antigen. The two FTS-positive individuals, one male from Abrekum aged 38 years, and a female from Obiri aged 76 years, reported being treated in the last MDA round 10 years ago. The prevalence in these communities was 1/75 (1.33%; 95% CI, 0.03%-7.21%) in Abrekum and 1/93 (1.08%; 95% CI, 0.03%-5.85%) in Obiri. The participant in Obiri consented to night blood collection, but no mf was observed. Regarding the last treatment status, a significant proportion of participants (71.2%, n = 373) reported having received treatment during the last MDA round, while 28.8% (n = 151) indicated they had not received treatment.

### Entomological surveys

A total of 763 mosquitoes were collected from the seven study communities using the pyrethrum spray collections. Of these, 475 (62.3%) were *Anopheles spp*., 23 (3.0%) were *Mansonia spp.*, 208 (27.3%) were *Culex spp*., and 57 (7.4%) were *Aedes spp*. The *Anopheles spp*. caught comprised of 70.9% (337/475) *An. gambiae* s.l. and 29.1% (138/475) *An. funestus*. Ayesuano had the largest share of sampled mosquitoes, accounting for 20.0% (153) of the total. Mankoadze had the highest number of *Culex* mosquitoes, accounting for 17.6% (134) of the total. *An. gambiae*, which is the main vector of lymphatic filariasis in Ghana, was the most frequently sampled (44.2%, n = 337) mosquito species (Table 1).

**Table 1:** Distribution of mosquito species collected across the study communities.

Community	Anophelines		Culicines			Sampled mosquitoes (%)
	*An. gambiae*	*An. funestus*	*Culex*	*Mansonia*	*Aedes*	
Ayesuano	107	15	25	3	3	153 (20.0)
Abrekum	48	24	18	9	6	105 (13.8)
Hwida	43	17	18	1	24	103 (13.5)
Obiri	17	11	16	1	8	53 (6.9)
Fawomanye	54	28	19	3	1	105 (13.8)
Amanful	39	33	20	5	13	110 (14.4)
Mankoadze	29	10	92	1	2	134 (17.6)
Total	337	138	208	23	57	763 (100.0)

### Molecular identification of *An. gambiae* s.l

Of all *An. gambiae* s.l. collected from the seven communities, 25% (85/337) were subjected to molecular identification using PCR. Of these, 92.9% (79/85) successfully amplified and 7.1% (6/85) did not amplify. Out of the 79 that amplified, 89.9% (71/79) were identified as *An. gambiae* s.l. and 10.1% (8/79) were identified as *An. melas.* Of the *An. gambiae* s.l. specimens, 74.6% (53/71) were further identified as *An. coluzzii* (previously known as the M form) and 25.4% (18/71) as *An. gambiae* s.s (previously known as the S form).

### Infection and infectivity of mosquitoes

A total of 475 *Anopheles* and 208 *Culex* mosquitoes from all the study communities were dissected and examined for *W. bancrofti* infection. Infections (mf) were exclusively found in *An. gambiae* mosquitoes from Ayesuano community. Five out of 107 *An. gambiae* mosquitoes from Ayesuano were infected with an infection rate of 4.7% (95% CI, 2.2%-8.5%). Three of these infected mosquitoes harbored the infective L3 stage of *W. bancrofti* with an infectivity rate of 2.8% (95% CI, 0.9%-7.5%). Considering the total number of *An. gambiae* collected from all study communities (n = 337), the infection rate was 1.5% (95% CI, 0.8%-2.9%), while the infectivity rate was 0.9% (95% CI, 0.5%-2.3%). Infection and infectivity rates due to all *Anopheles* spp. (n = 475) were 1.1% (95% CI, 0.5%-2.6%) and 0.6% (95% CI, 0.2%-1.5%) respectively (Table 2). Among these infected mosquitoes, three first-stage (L1) larvae were found in two mosquitoes. Additionally, four second-stage (L2) larvae and three third-stage (L3) larvae were identified each in four, and three mosquitoes respectively (Table 3).

**Table 2:** Infection and infectivity rates of mosquitoes from study communities (dissection).

Community	Species	No dissected	No. with larvae (L3)	% Infected (95% CI)	% Infective (95% CI)
Ayesuano	*An. gambiae*	107	5 (3)	4.7 (2.2-8.5)	2.8 (0.9-7.5)
Ayesuano	*An. funestus*	15	0	0.0 (0.0-22.7)	0.0 (0.0-22.7)
Ayesuano	*Culex* spp.	25	0	0.0 (0.0-13.7)	0.0 (0.0-13.7)
Abrekum	*An. gambiae*	48	0	0.0 (0.0-7.6)	0.0 (0.0-7.6)
Abrekum	*An. funestus*	24	0	0.0 (0.0-14.3)	0.0 (0.0-14.3)
Abrekum	*Culex* spp.	18	0	0.0 (0.0-18.4)	0.0 (0.0-18.4)
Hwida	*An. gambiae*	43	0	0.0 (0.0-8.5)	0.0 (0.0-8.5)
Hwida	*An. funestus*	17	0	0.0 (0.0-20.9)	0.0 (0.0-20.9)
Hwida	*Culex* spp.	18	0	0.0 (0.0-18.4)	0.0 (0.0-18.4)
Obiri	*An. gambiae*	17	0	0.0 (0.0-20.9)	0.0 (0.0-20.9)
Obiri	*An. funestus*	11	0	0.0 (0.0-29.6)	0.0 (0.0-29.6)
Obiri	*Culex* spp.	16	0	0.0 (0.0-22.2)	0.0 (0.0-22.2)
Fawomanye	*An. gambiae*	54	0	0.0 (0.0-6.5)	0.0 (0.0-6.5)
Fawomanye	*An. funestus*	28	0	0.0 (0.0-12.5)	0.0 (0.0-12.5)
Fawomanye	*Culex* spp.	19	0	0.0 (0.0-18.7)	0.0 (0.0-18.7)
Amanful	*An. gambiae*	39	0	0.0 (0.0-9.2)	0.0 (0.0-9.2)
Amanful	*An. funestus*	33	0	0.0 (0.0-10.9)	0.0 (0.0-10.9)
Amanful	*Culex* spp.	20	0	0.0 (0.0-17.7)	0.0 (0.0-17.7)
Mankoadze	*An. gambiae*	29	0	0.0 (0.0-12.8)	0.0 (0.0-12.8)
Mankoadze	*An. funestus*	10	0	0.0 (0.0-30.9)	0.0 (0.0-30.9)
Mankoadze	*Culex* spp.	92	0	0.0 (0.0-3.9)	0.0 (0.0-3.9)
	***An. gambiae***	337	5 (3)	1.5 (0.8-2.9)	0.9 (0.5-2.3)
	***An. funestus***	138	0	0.0 (0.0-2.7)	0.0 (0.0-2.7)
	***Culex* spp.**	208	0	0.0 (0.0-3.9)	0.0 (0.0-3.9)
**Total**	***Anopheles spp.***	475	5 (3)	**1.1 (0.5-2.6)**	**0.6 (0.2-1.5)**

**Table 3:** The number of larval stages of *W. bancrofti* in infected *Anopheles gambiae* populations at Ayesuano.

Mosquito species ID	Number of larvae identified in infected mosquitoes		
	L1	L2	L3
AY-P1	**2**	**1**	**1**
AY-P2	**0**	**0**	**1**
AY-P3	**1**	**1**	**0**
AY-P4	**0**	**1**	**0**
AY-P5	**0**	**1**	**1**

### Pool screening PCR of mosquitoes

A total of 683 mosquitoes comprising 337 *An. gambiae*, 138 *An. funestus* and 208 *Culex* spp. were analyzed in pools (1-20 mosquitoes per pool) for *W. bancrofti* infection. Pools were analyzed based on species and community (Table 4). A total of 57 pools; 28 *An. gambiae* pools, 13 *An. funestus* pools and 16 *Culex* pools were analyzed. Infected mosquitoes were highly clustered at the community level, with all positive pools coming from only two communities and an estimated intra-cluster correlation of 0.2 (95% CI, 0.01-0.75) for all *Anopheles* species. Six *An. gambiae* pools from Ayesuano tested positive for *W. bancrofti* out of a total of 12 pools (107 mosquitoes). Additionally, a single positive pool was identified from Hwida and this was also an *An. gambiae* pool. After adjusting for clustering at the community level, the estimated infection prevalence for the mosquito species were: *Anopheles* spp. 2.5% (95% CI, 0.4-23.5%), *An. gambiae* 3.1% (95% CI, 0.5-24.0%), *An. funestus* 0.0% (95% CI, 0.0-4.3%), and *Culex* spp. 0.0% (95% CI, 0.0%, 3.7%). The estimated prevalence in *An. gambiae* was 6.5% (95% CI, 3.3-12.7%) in Ayesuano and 2.4% (95% CI, 0.2-13.3%) in Hwida (Table 4).

**Table 4:** Estimated prevalence of infection in mosquitoes from study communities.

Community	Species	No of mosquitoes analyzed	No of pools	Pools Positive	Prevalence (95% CI)^a^
Ayesuano	*An. gambiae*	107	12	6	6.5 (3.3-12.7)
Ayesuano	*An. funestus*	15	1	0	0.0 (0.0-20.4)
Ayesuano	*Culex* spp.	25	6	0	0.0 (0.0-13.3)
Abrekum	*An. gambiae*	48	3	0	0.0 (0.0-7.4)
Abrekum	*An. funestus*	24	5	0	0.0 (0.0-13.8)
Abrekum	*Culex* spp.	18	1	0	0.0 (0.0-17.6)
Hwida	*An. gambiae*	43	5	1	2.4 (0.2-13.3)
Hwida	*An. funestus*	17	1	0	0.0 (0.0-18.4)
Hwida	*Culex* spp.	18	1	0	0.0 (0.0-17.6)
Obiri	*An. gambiae*	17	1	0	0.0 (0.0-18.4)
Obiri	*An. funestus*	11	1	0	0.0 (0.0-25.9)
Obiri	*Culex* spp.	16	1	0	0.0 (0.0-19.4)
Fawomanye	*An. gambiae*	54	3	0	0.0 (0.0-6.6)
Fawomanye	*An. funestus*	28	2	0	0.0 (0.0-12.1)
Fawomanye	*Culex* spp.	19	1	0	0.0 (0.0-16.8)
Amanful	*An. gambiae*	39	2	0	0.0 (0.0-9.0)
Amanful	*An. funestus*	33	2	0	0.0 (0.0-10.4)
Amanful	*Culex* spp.	20	1	0	0.0 (0.0-16.1)
Mankoadze	*An. gambiae*	29	2	0	0.0 (0.0-11.7)
Mankoadze	*An. funestus*	10	1	0	0.0 (0.0-27.8)
Mankoadze	*Culex* spp.	92	5	0	0.0 (0.0-4.0)
	***An. gambiae***	337	28	7	3.1 (0.5-24.0)
	***An. funestus***	138	13	0	0.0 (0.0-4.3)
	***Culex* spp.**	208	16	0	0.0 (0.0-3.7)
**Total**	***Anopheles* spp.**	475	41	7	**2.5 (0.4-23.5)**

a Estimates for each community use Firth's bias correction for the point estimate and score confidence intervals corrected for skew [23]. Estimates for the district are adjusted for clustering at the community level and use the posterior median for the point estimate and symmetric credible intervals [26].

### Estimation of transmission indices

The biting rates observed across the seven communities were generally low, with some variations. However, Ayesuano recorded a relatively higher biting rate (BR = 0.70, ABR = 255.92), indicating a greater frequency of mosquito bites in this community compared to the other communities (Table S2). In contrast, Abrekum, Hwida, Fawomanye, Amanful and Mankoadze recorded similar and lower biting rates, reflecting reduced exposure to mosquito bites in these communities. Obiri recorded the lowest biting rate of 0.18 bites/person/night. Notably, Ayesuano also posed an ongoing risk of transmission, with 2.46% of captured mosquitoes found to be infective for *W. bancrofti*. This resulted in an Annual Infective Biting Rate (AIBR) of 6.30, implying that a resident from this community is exposed to approximately 630 bites from infective mosquitoes in a year. In contrast, the other communities had an AIBR of 0.0, indicating no risk of infection.

Since no infective mosquitoes were found in other communities except Ayesuano, the infectivity rates (IR) were zero, and as a result, the ATP values were also zero, signifying a low risk of transmission in the district. Each infective mosquito was found to harbour only one L3 larva, corresponding to a worm load (WL) of 1.0. This worm load, coupled with the AIBR, contributed to an Annual Transmission Potential (ATP) of 6.30 in Ayesuano (Table S2).

*Anopheles funestus* captured in this study were not found with *W. bancrofti* larvae resulting in zero values for IR, AIBR, and ATP. While *An. gambiae* showed a very low ATP of 0.93 (Table S3), the infectivity rate in this species was 0.009 for the entire district. However, when looking at all Anopheles mosquitoes, the ATP was 0.88 for the entire district.

Prior to MDA in the Gomoa District, the mf prevalence in the human population was 4.6%, and the CFA prevalence was 8.7% [9]. Ten years after MDA cessation, mf prevalence was 0.0%, and the antigen prevalence also decreased to 0.4% (Table S4).

The AIBR significantly changed over time. Prior to MDA in 2003, *An. gambiae* and *An. funestus* had AIBR rates of 119.8 and 113.7, respectively [9]. The ATP was also high; 311.4 infective larvae/person/year for *An. gambiae* and 153.5 infective larvae/person/year for *An. funestus*. Ten years post-MDA cessation*,* AIBR and ATP values are very low. *An. gambiae* recorded similar AIBR and ATP values of 0.93, while *An. funestus* was 0.0.

## Discussions

To assess the current transmission status of LF in the Gomoa West district of Ghana, where MDA has stopped for 10 years now, a cross-sectional population survey and entomological surveys were conducted in seven communities within the district. The cross-sectional population survey using the FTS revealed a very low CFA prevalence of 0.4%. The low CFA prevalence observed in the study district is consistent with previous studies that have reported a decline in the prevalence of LF in areas that have implemented MDA [20]. The Gomoa West district received 12 annual rounds of MDA from 2003 until 2014 when the district ceased MDA after passing the TAS. Ten years after MDA cessation, the CFA results indicate that LF prevalence remains low.

The entomological surveys revealed a higher number of *An. gambiae s.l*., the primary vector for LF in Ghana [21]. *An. melas*, a sibling species within the *An. gambiae* complex was identified at Ayesuano, Hwida, and Amanful. Amuzu et al. [22] also found *An. melas* at Hwida exhibiting different vectoral capacities in the then Gomoa district of Ghana. In this study, smaller numbers of *An. melas* were identified compared to *An. gambiae s.s*. Furthermore, molecular species identification of the *An. gambiae* s.l indicated a higher number of *An. coluzzii* (formerly the M form) across all seven communities studied. The M form is known to be a more efficient vector of LF compared to the S form [23].

From this study high infection rates by pool screening were reported in the *An. gambiae* from Ayesuano (6.5%, 95% CI, 3.3, 12.7%) and Hwida (2.4%, 95% CI, 0.2, 13.3%). Together, the infection prevalence for the entire district was 2.5% (95% CI, 0.4%, 23.5%), exceeding the 1% provisional threshold established by the WHO for *Anopheles*-transmitted LF [24]. The detection of the L3 of *W. bancrofti* in infected *An. gambiae* mosquitoes suggest ongoing transmission in the district. The infectivity rate in *An. gambiae* was 0.9% (95% CI, 0.5%, 2.3%), which is notably high, underscores the significance of *An. gambiae* in the ongoing transmission within the district. Despite these, the ATP was very low, begging the question of whether transmission is widespread in the district or limited to specific communities.

While the positive humans and mosquitoes are not from the same communities, and no mfs were detected in humans, WHO provides provisional thresholds (Aedes spp. - 0.1%; Anopheles spp. - 1.0%; Culex spp. - 0.5%; Mansonia spp. - 0.5%) below which transmission can be considered to be interrupted.[24] It has also been shown by other studies [[25], [26], [27]] that molecular xenomonitoring can be used for monitoring recrudescence of infection in post-MDA and validation phases when the infection is at a level lower than that detectable by Ag or microfilaria testing. Thus, the high pool screening prevalence of 2.5% in the district is above the 1% threshold recommended by WHO and as such indicative of a potential resurgence and transmission.

In the Gomoa District, the pre-MDA phase was marked by high transmission indices, including parasite prevalence, infection rate, and annual transmission potential, indicating a significant burden of LF [9]. Ten years after the cessation of MDA, the biting rates and ATP values in the district remain low. This suggests that the overall mosquito-human transmission dynamics have been greatly suppressed. However, despite these reduced transmission indices, the infection and infectivity rates within the mosquito vectors remain above the WHO provisional threshold of 1% in Anopheles-transmitted areas [24]. While transmission has decreased, the infection in mosquitoes indicates that there are still infected individuals capable of sustaining the transmission of the parasite. This ongoing transmission is likely sustained by small pockets of infected individuals or persistent mosquito activity in specific areas, even though microfilariae were not detected in the human population and the antigen prevalence was very low. This study highlights the significance of molecular xenomonitoring as an essential tool for post-MDA and post-validation surveillance [26]. The Second Edition of the WHO manual titled “Monitoring and Epidemiological Assessment of Mass Drug Administration in the Global Programme to Eliminate Lymphatic Filariasis: A Manual for National Elimination Programmed” (In press) features a significant expansion of the chapter on surveillance. This updated version provides recommendations for conducting Postvalidation Surveillance (PVS) - conducted any time after TAS3. It suggests that PVS activities should utilize a combination of at least two out of four platforms, one of which is Molecular Xenomonitoring (MX).

A study using three well-established mathematical models, indicates that there remains a risk of LF resurgence following the cessation of MDA although this resurgence may take time to occur [28]. Similarly, Smith et al. [29] found that the risk of resurgence increases over time following the cessation of MDA. Field studies also support these findings [28]. The potential for resurgence underscores the critical importance of post-MDA surveillance. Without sustained and intensive monitoring, there is a substantial risk that the progress achieved through years of MDA could be undone, potentially leading to the resurgence of LF as an endemic disease [30].

Singh and Michael [31] highlighted the variability in the time required to eliminate LF parasites in response to MDA and other strategies, particularly when vector control is incorporated. Their model reveals that including vector control measures not only reduces the duration of intervention needed to achieve elimination but also decreases the risk of LF resurgence [31]. Jambulingam et al. [32] support this by confirming that the critical thresholds used as endpoints for MDA will depend on local transmission conditions.

In the pre-MDA study [9], the MBR ranged from 311-6116, compared to the MBR ranges in this study of 5.34-21.39. In this study, Ayesuano where mf was identified through dissection had the highest MBR of 21.39, representing less than 1 bite per person per day. This drastic decline in MBR could be attributed to the impacts of climate change [33]. However, this could also be due to the use of treated nets and other mosquito protection measures resulting in few mosquitoes resting indoors, as these indoor vector control measures have been shown to result in mosquito behavior modifications with many biting indoors and resting outdoors or switching to outdoor biting [34]. While bednet usage was observed in some houses, the coverages were unfortunately not recorded in the study. However, it is also likely that transmission occurs more outdoors than indoors, as observed in malaria following vector control interventions [34].

Some limitations were encountered in this study. One significant limitation was the sample size in the human and vector population analyzed. In the human studies, only 524 participants from seven communities were enrolled which may not fully represent the entire population of the Gomoa West District. Additionally, some communities, such as Ayesuano and Mankoadze, were underrepresented compared to others. This underrepresentation occurred as participants were chosen based on their availability and willingness to take part in the study, potentially leading to prevalence estimates that may not be reflective of the larger population. It should also be noted that the sampling approach of convenient and centralized testing means that mostly individuals who are amenable to LF interventions (indicated by the high proportion who reported to have taken part in the last MDA) took part in the study, potentially leaving out those who for various reasons refuse participation or are unavailable. Further, communities with previously high burdens were selected for sampling to increase the chance of finding a signal, but consequently, overall prevalence in humans and vectors was likely to be higher in the selected communities than in the broader Gomoa West District. Low numbers of mosquitoes were collected over the six-month collection period. Combined with a high degree of clustering at the community level, this led to uncertain estimates of district-level prevalence. We were unable to account for the clustering of infection at the household level as some pools combined mosquitoes from different households due to small catches. There was some discordance between the results of dissection and PCR testing, also observed in the earlier studies [9], suggesting that both methods have imperfect sensitivity and therefore somewhat underestimate true infection/infective rates. Standard methods of estimating prevalence from pooled samples assume that mosquitoes are placed in pools independent of their infection status. However, in our protocol, infected mosquitoes were more likely to be placed in single pools, which may have also somewhat biased our estimates. Another limitation was the reliance on self-reported data and questionnaires to assess participation and compliance with the MDA 10 years ago. This is prone to recall bias, potentially affecting the accuracy of the information collected. Lastly, the potential for cross-border transmission may exist. However, the study did not assess the movement of participants, their migration history or cross-border transmission.

In conclusion, this study detected *W. bancrofti* infections in the human and *Anopheles* populations in the Gomoa West district of Ghana, where MDA ceased 10 years ago. The observed infection rate in *Anopheles spp*. exceeds the 1% threshold set by the WHO [24], suggesting a risk of ongoing transmission and potential resurgence of LF in the district. The updated WHO manual (In press) states that if the number of positive mosquito pools exceeds the species-specific threshold, programs may either conduct a targeted LF survey or directly proceed to two rounds of targeted treatment. As such, these findings suggest the need for interventions such as MDA and/or vector control in the district. However, it will be important to confirm these through district-wide assessments to determine whether interventions should be implemented in the entire district or targeted to particular sub-districts or communities.

## Declarations of competing interest

The authors have declared that no competing interests exist.

## Appendix Supplementary materials

Image, application 1

## References

[1] Ottesen E.A (2000). Editorial: the global programme to eliminate lymphatic filariasis. Trop Medi Int Health.

[2] World Health Organization (2024). Global Programme to eliminate lymphatic filariasis: progress report, 2023. Weekly Epidemiol Records.

[3] World Health Organization Regional Office for Africa. Expanded special project for the elimination of neglected tropical diseases (ESPEN) portal. [cited 19 Dec 2024]. available: https://espen.afro.who.int/diseases/lymphatic-filariasis Dataset

[4] World Health Organization (2011).

[5] Santoso, Yahya, Supranelfy Y., Suryaningtyas N.H., Taviv Y., Yenni A. (2020). Risk of recrudescence of lymphatic filariasis after Post-MDA surveillance in Brugia malayi endemic Belitung District, Indonesia. Korean J Parasitol.

[6] Rebollo M.P., Mohammed K.A., Thomas B., Ame S., Ali S.M., Cano J. (2015). Cessation of mass drug administration for lymphatic filariasis in Zanzibar in 2006: was transmission interrupted?. PLoS Negl Trop Dis.

[7] Biritwum N.K., Frempong K.K., Verver S., Odoom S., Alomatu B., Asiedu O. (2019). Progress towards lymphatic filariasis elimination in Ghana from 2000-2016: analysis of microfilaria prevalence data from 430 communities. PLoS Negl Trop Dis.

[8] Biritwum N.-K., Yikpotey P., Marfo B.K., Odoom S., Mensah E.O., Asiedu O. (2016). Persistent “hotspots” of lymphatic filariasis microfilaraemia despite 14 years of mass drug administration in Ghana. Trans R Soc Trop Med Hyg.

[9] Aboagye-Antwi F., Kwansa-Bentum B., Dadzie S.K., Ahorlu C.K., Appawu M.A., Gyapong J. (2015). Transmission indices and microfilariae prevalence in human population prior to mass drug administration with ivermectin and albendazole in the Gomoa District of Ghana. Parasit Vectors.

[10] World Health Organization (2000).

[11] Weil G.J., Curtis K.C., Fakoli L., Fischer K., Gankpala L., Lammie P.J. (2013). Laboratory and field evaluation of a new rapid test for detecting Wuchereria bancrofti antigen in human blood. Am J Trop Med Hyg.

[12] McMahon J.E., Marshall T.F.D.C., Vaughan J.P., Abaru D.E (1979). Bancroftian filariasis: a comparison of microfilariae counting techniques using counting chamber, standard slide and membrane (nuclepore) filtration. Ann Trop Med Parasitol.

[13] Gillies M.T., De Meillon B (1968).

[14] Ramzy R.M., Farid H.A., Kamal I.H., Ibrahim G.H., Morsy Z.S., Faris R. (1997). A polymerase chain reaction-based assay for detection of Wuchereria bancrofti in human blood and Culex pipiens. Trans R Soc Trop Med Hyg.

[15] Fanello C., Santolamazza F., Della Torre A (2002). Simultaneous identification of species and molecular forms of the Anopheles gambiae complex by PCR-RFLP. Med Vet Entomol.

[16] Biggerstaff B. (2005). PooledInfRate software. Vector Borne Zoonotic Dis.

[17] Ebert T.A., Brlansky R., Rogers M (2010). Reexamining the pooled sampling approach for estimating prevalence of infected insect vectors. Ann Entomol Soc Am.

[18] Moustafa M.A.M., Mohamed W.M.A., Lau A.C.C., Chatanga E., Qiu Y., Hayashi N. (2020). R A language and environment for statistical computing, R Foundation for Statistical. Computing.

[19] McLure A., O'Neill B., Mayfield H., Lau C., McPherson B (2021). PoolTestR: an R package for estimating prevalence and regression modelling for molecular xenomonitoring and other applications with pooled samples. Envir Model Software.

[20] Simonsen P.E., Derua Y.A., Magesa S.M., Pedersen E.M., Stensgaard A.S., Malecela M.N. (2014). Lymphatic filariasis control in Tanga region, Tanzania: status after eight rounds of mass drug administration. Parasit Vectors.

[21] de Souza D., Kelly-Hope L., Lawson B., Wilson M., Boakye D (2010). Environmental factors associated with the distribution of Anopheles gambiae s.s in Ghana; an important vector of lymphatic filariasis and malaria. PLoS One.

[22] Amuzu H., Wilson M.D., Boakye D.A (2010). Studies of Anopheles gambiae s.l (Diptera: culicidae) exhibiting different vectoral capacities in lymphatic filariasis transmission in the Gomoa district, Ghana. Parasit Vectors.

[23] Hunter J.M (1992). Elephantiasis: a disease of development in North East Ghana. Soc Sci Med.

[24] World Health Organization. The role of polymerase chain reaction techniques for assessing lymphatic filariasis transmission. Geneva; 2009.

[25] Pryce J., Reimer L.J (2021). Evaluating the Diagnostic Test Accuracy of Molecular Xenomonitoring Methods for Characterizing Community Burden of Lymphatic Filariasis. Clin Infect Dis.

[26] Subramanian S., Jambulingam P., Krishnamoorthy K., Sivagnaname N., Sadanandane C., Vasuki V. (2020). Molecular xenomonitoring as a post-MDA surveillance tool for global programme to eliminate lymphatic filariasis: field validation in an evaluation unit in India. PLoS Negl Trop Dis.

[27] Irish S.R., Al-Amin H.M., Paulin H.N., Mahmood A.S.M.S., Khan R.K., Muraduzzaman A.K.M. (2018). Molecular xenomonitoring for Wuchereria bancrofti in Culex quinquefasciatus in two districts in Bangladesh supports transmission assessment survey findings. PLoS Negl Trop Dis.

[28] Prada J.M., Davis E.L., Touloupou P., Stolk W.A., Kontoroupis P., Smith M.E. (2020). Elimination or Resurgence: modelling Lymphatic Filariasis After Reaching the 1% Microfilaremia Prevalence Threshold. J Infect Dis.

[29] Smith M.E., Singh B.K., Irvine M.A., Stolk W.A., Subramanian S., Hollingsworth T.D. (2017). Predicting lymphatic filariasis transmission and elimination dynamics using a multi-model ensemble framework. Epidemics.

[30] Irvine M.A., Kazura J.W., Hollingsworth T.D., Reimer L.J (2018). Understanding heterogeneities in mosquito-bite exposure and infection distributions for the elimination of lymphatic filariasis. Proc Biol Sci.

[31] Singh B.K., Michael E (2015). Bayesian calibration of simulation models for supporting management of the elimination of the macroparasitic disease, Lymphatic Filariasis. Parasit Vectors.

[32] Jambulingam P., Subramanian S., De Vlas S.J., Vinubala C., Stolk W.A (2016). Mathematical modelling of lymphatic filariasis elimination programmes in India: required duration of mass drug administration and post-treatment level of infection indicators. Parasit Vectors.

[33] Giesen C., Roche J., Redondo-Bravo L., Ruiz-Huerta C., Gomez-Barroso D., Benito A. (2020). The impact of climate change on mosquito-borne diseases in Africa. Pathog Glob Health.

[34] Thomsen E.K., Koimbu G., Pulford J., Jamea-Maiasa S., Ura Y., Keven J.B. (2017). Mosquito behavior change after distribution of bednets results in decreased protection against malaria exposure. J Infect Dis.

