# Supplementary material for: Lymphatic filariasis transmission 10 years after stopping mass drug administration in the Gomoa west district of Ghana
Source: Int J Infect Dis. 2025 Mar;152:None. doi: 10.1016/j.ijid.2025.107790 (PMC11873683; doi:10.1016/j.ijid.2025.107790)
Supplement: Supplementary file 1 [file mmc1.docx]

**Supplementary Materials**

**Table of Contents**

[Table S1: Cross-sectional survey results 2](#_Toc185421671)

[Table S2: Estimation of community transmission indices 3](#_Toc185421672)

[Table S3: Estimation of district transmission indices by vector species. 4](#_Toc185421673)

[Table S4: Comparison of transmission indices before MDA and 10 years after stopping MDA 5](#_Toc185421674)

## **Table S1:** Cross-sectional survey results

| **Community** | **No. tested** | **Age range (yrs)** | **No. Positive** | **Prevalence (95% CI)** |
| --- | --- | --- | --- | --- |
| Ayesuano | 51 | 18-82 | 0 | 0.00% (0.00% - 6.98%) |
| Fawomanye | 74 | 18-74 | 0 | 0.00% (0.00% - 4.86%) |
| Amanful | 82 | 18-82 | 0 | 0.00% (0.00% - 4.40%) |
| Obiri | 93 | 18-85 | 1 | 1.08% (0.03% - 5.85%) |
| Hwida | 105 | 18-78 | 0 | 0.00% (0.00% - 3.45%) |
| Mankoadze | 44 | 18-83 | 0 | 0.00% (0.00% - 8.04%) |
| Abrekum | 75 | 18-87 | 1 | 1.33% (0.03% - 7.21%) |

## **Table S2:** Estimation of community transmission indices

| **Community** | **No of *Anopheles* spp.** | **Total No of sleepers** | **No of catches** | **BR** | **MBR** | **ABR** | **IR**  **(95% CI)** | **AIBR (95% CI)** | **WL** | **ATP**  **(95% CI)** |
| --- | --- | --- | --- | --- | --- | --- | --- | --- | --- | --- |
| **Ayesuano** | 122 | 174 | 136 | 0.70 | 21.39 | 255.92 | 0.0246 (0.005-0.070) | 6.30 (1.28-17.91) | 1.00 | 6.30 (1.28-17.91) |
| **Abrekum** | 72 | 183 | 141 | 0.39 | 12.00 | 143.61 | 0.00 (0-0.049) | 0.00 (0-7.04) | NA | NA |
| **Hwida** | 60 | 157 | 148 | 0.38 | 11.66 | 139.49 | 0.00  (0-0.060) | 0.00  (0-8.37) | NA | NA |
| **Obiri** | 28 | 160 | 155 | 0.18 | 5.34 | 63.88 | 0.00  (0-0.012) | 0.00  (0-0.77) | NA | NA |
| **Fawomanye** | 82 | 176 | 147 | 0.47 | 14.21 | 170.06 | 0.00  (0-0.044) | 0.00  (0-7.48) | NA | NA |
| **Amanful** | 72 | 149 | 132 | 0.48 | 14.74 | 176.38 | 0.00  (0-0.050) | 0.00  (0-8.82) | NA | NA |
| **Mankoadze** | 39 | 189 | 143 | 0.21 | 6.29 | 75.32 | 0.00  (0-0.090) | 0.00  (0-6.78) | NA | NA |

Abbreviations and definitions: BR (Biting rate) = (no of mosquitoes / no of sleepers); MBR (Monthly Biting Rate) = BR x 30.5 days; ABR (Annual Biting Rate) = BR x 365 days; IR (Infectivity Rate) = (no of L3-postive mosquitoes / Total no of mosquitoes collected) x 100 ; AIBR (Annual Infective Biting Rate) = ABR x IR; WL (Worm load) = Total no of L3 larvae / Total no of mosquitoes carrying L3; ATP (Annual Transmission Potential) = AIBR x WL. **Note that these are estimates based on the PSC method.**

## Table S3: Estimation of district transmission indices by vector species.

| **Mosquitoes spp.** | **No of mosquitoes** | **No of sleepers** | **No of catches** | **BR** | **MBR** | **ABR** | **IR**  **(95% CI)** | **AIBR**  **(95% CI)** | **WL** | **ATP**  **(95% CI)** |
| --- | --- | --- | --- | --- | --- | --- | --- | --- | --- | --- |
| *An. gambiae* s.l. | 337 | 1188 | 1002 | 0.28 | 8.65 | 103.54 | 0.009  (0.002-0.026) | 0.93  (0.52-2.38) | 1.00 | 0.93  (0.21-2.69) |
| *An. funestus* | 138 | 1188 | 1002 | 0.12 | 3.54 | 42.40 | 0  (0-0.026) | 0  (0-1.14) | NA | NA |
| *All Anopheles* | 475 | 1188 | 1002 | 0.40 | 12.19 | 145.94 | 0.006  (0.002-0.015) | 0.88  (0.15-1.09) | 1.0 | 0.88 (0.15-1.09) |

Abbreviations and definitions: BR (Biting rate) = (no of mosquitoes / no of sleepers); MBR (Monthly Biting Rate) = BR x 30.5 days; ABR (Annual Biting Rate) = BR x 365 days; IR (Infectivity Rate) = (no of L3-postive mosquitoes / Total no of mosquitoes collected) x 100 ;
AIBR (Annual Infective Biting Rate) = ABR x IR; WL (Worm load) = Total no of L3 larvae / Total no of mosquitoes carrying L3;
ATP (Annual Transmission Potential) = AIBR x WL. **Note that these are estimates based on the PSC method.**

Table S4: Comparison of transmission indices before MDA and 10 years after stopping MDA.

| Transmission Indicator | Before MDA [12] | 10 years After MDA Cessation  (this study) |
| --- | --- | --- |
| Mosquitoes collected | 17,784 | 763 |
| Mosquito collection time frame | 4 nights/month over 6 months | Once a month over 6 months |
| Mosquito collection method | Human landing collections | Pyrethrum spray collections |
| Individuals tested | 941(mf); 861 (Ag) | 524 |
| Prevalence | 4.6% mf prevalence; 8.7% CFA prevalence | 0.0% mf prevalence; 0.38% CFA prevalence |
| MBR ranges | 311 – 6116 | 5.34 – 21.39 |
| AIBR* | *An. gambiae*: 119.8, *An. funestus*: 113.7 | *An. gambiae*: 0.93, *An. funestus*: 0.0 |
| ATP* | *An. gambiae*: 311.4, *An. funestus*: 153.5 | *An. gambiae*: 0.93, *An. funestus*: 0.0 |
| Infection rate* | *Anopheles spp*: 0.7% (CI: 0.5-0.9) | *Anopheles spp*: 1.1% (CI: 0.5-2.6) |
| Infectivity rates* | *Anopheles spp*: 0.3% (CI: 0.2-0.4) | *Anopheles spp*: 0.6% (CI: 0.2-1.5) |

***** estimated based on mosquito dissection data
